# The role of risk perception in willingness to respond to the 2014–2016 West African Ebola outbreak: a qualitative study of international health care workers.

**DOI:** 10.1186/s41256-017-0042-y

**Published:** 2017-08-07

**Authors:** Stephanie Gee, Morten Skovdal

**Affiliations:** 1 School of Health and Related Research, University of Sheffield, 30 Regent St, Sheffield, S1 4DA UK; 2 Department of Public Health, University of Copenhagen, Øster Farimagsgade 5, 1014 Copenhagen, Denmark

**Keywords:** Risk perception, Health care workers, Ebola, Infectious disease outbreaks, West Africa

## Abstract

**Background:**

The 2014–2016 West Africa Ebola Virus Disease (EVD) outbreak was an unprecedented public health event, and in addition to claiming over 11,000 lives, it resulted in the deaths of more healthcare workers than any outbreak in recent history. While a cadre of willing and able health workers is essential for an effective epidemic response, health workforce capacity in times of crisis may be significantly impacted by how risks are perceived by health staff. This study aimed to explore how risk perceptions influenced healthcare workers’ willingness to respond during this outbreak.

**Methods:**

Through in-depth interviews with 11 front-line international health care workers who chose to respond to the West Africa outbreak, this qualitative study explores how perceptions of risk developed and subsequently mediated the decision to respond to the outbreak. Data was thematically organized using NVivo 10.

**Results:**

We found that numerous individual and social-level factors played a role in modifying risk perception in health workers. Institutional trust emerged as a key risk attenuator, as did past experience, self-efficacy, duty of care, humanitarian ethos, and cognitive heuristics. Feelings of risk were amplified by infections of co-workers, and risk perceptions of family members and the public, which were mainly informed by media reports, also hampered willingness to respond in some cases.

**Conclusions:**

Understanding the risk perceptions of health workers, institutions, and the public, while complex and interdependent, are each crucial to understand for an effective public health response to epidemics, and as such should be taken into consideration in future program planning and research.

## Background

The 2014–2016 West African epidemic of Ebola Virus Disease (EVD) has been described as ‘the most severe acute public health emergency seen in modern times’ [1] and the impact it had on the three most affected countries – Liberia, Sierra Leone and Guinea – has been catastrophic, not least to their health-care infrastructure and workforce [2]. A combination of factors, including the emergence of an unfamiliar disease in settings with degraded infrastructure and minimal public health capacity, has contributed to high rates of health worker infections. Health care workers in West Africa were shown to be at 21–32 times higher risk of contracting the disease compared to the general population, and by the end of the outbreak 881 health workers had become infected and 513 had died from the virus [3]. Due to the overwhelming nature of the epidemic, numerous calls for international medical help were made during the peak of the crisis, however according to a World Health Organisation (WHO) press release, ‘The loss of so many doctors and nurses has made it difficult for WHO to secure support from sufficient numbers of foreign medical staff’ [4]. The continual need to recruit and train international medical staff to support local health workers became an important focus for international agencies throughout the outbreak.

The significant need for, and risks to, healthcare workers raises many important questions about risk perception and its influence on their willingness to respond during infectious disease outbreaks: What factors attenuate and amplify perceptions of risk in front-line health staff? What are the characteristics and perceptions of successful responders, and what can be learned from them? How can we support a willing health workforce in advance of the next outbreak? Despite its critical importance few studies have explored the risk perceptions of international healthcare workers who volunteered to respond to the EVD outbreak. This qualitative study explores the dynamics of risk perception as it relates to willingness to respond in order to elucidate key factors that may support and inform a successful response effort in future outbreaks.

### Fear of contagion and willingness of health staff to respond during disease outbreaks

While there is both a need and an expectation that health care workers will be available to provide care in the event of a large-scale emergency, a number of studies suggest that there are significant limits to health workers’ willingness to respond. Historically, hesitation or refusal to provide care has been seen during the early years of the human immunodeficiency virus (HIV) [5, 6] and during the severe acute respiratory syndrome (SARS) outbreak [7]. The nature of the risk event appears to play a role in willingness to respond to emergencies with the fear of contagion of self and family placing willingness to respond to infectious disease outbreaks lower than for any other kind of large-scale emergency (e.g., snow storm, environmental disaster, or chemical incident) [8]. In fact, such limits to willingness may have serious implications for response capacities. A few studies have addressed this issue in the United States. A survey of 428 health care workers (HCWs) looking at willingness to respond to a hypothetical EVD patient in New York found that 25.1% of respondents thought it was ethical to refuse care to EVD patients, and 25.9% were ‘somewhat’ or ‘very’ unwilling to care for such a patient [9]. Only 44% of respondents felt their hospital was sufficiently prepared to deal with such a case, and 16.8% worried ‘quite often’ or ‘all the time’ about contracting EVD from a patient. It is also notable that the degree of concern about potentially exposing friends and family to EVD was 90% even when asymptomatic, while those worried about self-contagion were only 16.8%. Multivariate analysis found this concern for family predictive of unwillingness to provide care with an odds ratio of 11.1 [9].

Similarly, unwillingness to respond also has been demonstrated in studies around pandemic influenza. Studies exploring US health workers responses to a hypothetical influenza pandemic found that nearly half of all local public health department workers were unlikely to report for duty during an outbreak and up to 32% of hospital workers were unlikely to respond [10, 11]. In a multivariate analysis of survey results, the likelihood of reporting to work was significantly associated with a number of individual level factors such as the perception of the importance of, and familiarity with, one’s role in the response, level of knowledge of pandemic events, feeling psychologically prepared, feeling safe at work, and family preparedness [10, 11]. The study of public health workers found 66% of respondents perceived that they would be at personal risk of contagion while performing their duties. The confidence the staff had in their personal safety was associated with the perception of existing knowledge about the impact of the pandemic (OR 4.1 CI 2.3–7.6); family preparedness (OR 2.5; CI 1.4–4.3); perception that the health department was providing timely information (OR 5.4; CI 2.7–10.7), amongst others [10]. Similar findings were observed in a comparable study in India [12].

The above studies provide valuable insights but are limited by the hypothetical nature of their designs - asking health workers how they ‘would’ respond given certain scenarios rather than exploring these phenomena during actual outbreaks. Nonetheless, they suggest that the reduction of health workforce capacity may be significant.

There are only few qualitative studies exploring the willingness of health workers to respond to infectious disease outbreaks. A study by Gershon et al. [13] of the experiences of American health workers who volunteered to work in the 2014–2016 Ebola outbreak found many were motivated by a belief their skills were needed, an ethical obligation, a commitment to social justice, and having past experience in humanitarian crises. While they were concerned about the chance of infection, they also were impacted by the fear of family and friends, which led some to secrecy about their upcoming mission. A qualitative study by Ives et al. [14] exploring the willingness of health staff to respond to a hypothetical influenza pandemic in the UK found a number of enabling and prohibiting factors. Staff were motivated by a sense of obligation or duty of care; and barriers were related to giving priority to the health of family members, lack of trust in the National Health Service, lack of information on risks and role expectations during the outbreak, and feelings that administrators did not take concerns of front-line staff seriously.

The 2014–2016 West Africa EVD outbreak provides an important opportunity to further explore the factors mediating the willingness of international health workers to respond to the most deadly infectious disease outbreak in recent history. This study aims to explore such experiences, making use of risk perception theory to help unpack factors that influenced willingness to respond in a group of international health care workers.

### Risk perception theory

Risk perception theory can aid in exploring the reactions of individuals and societies to conditions of risk. Risk perception studies have shown that people do not utilize a simple objective weighting of probabilities when deciding which risks to pay attention to, fear, or avoid. Rather, risk perception is an abstract and socially constructed phenomenon with responses to risk events often difficult to predict: minor risks may become amplified to extreme levels, while other more deadly risks may be generally ignored [15]. Kasperson’s social amplification of risk framework (SARF) helps elucidate how a given risk event merges with psychological, social and cultural processes to amplify or attenuate feelings of risk [16]. This framework usefully highlights factors that influence risk perception, which include examining the characteristics of the risk event itself - particularly how ‘dreaded’ and ‘unknown’ it is, as well as how it is interpreted and communicated by social actors such as institutional stakeholders, traditional and social media, or government. These socially constructed risk messages are subsequently interpreted and acted on by the individual, dependent upon one’s attention filter, personal characteristics and attitudes [16]. Further, cognitive heuristics - sets of inferential rules that people employ to make judgments in conditions of uncertainty – also play a role in how risks are perceived by the individual [15]. For example, the ‘affect heuristic’ has shown how emotional responses to a risk event can increase or decrease feelings of risk [17] and it has also been shown that quick, emotional impressions often precede and guide ‘rational’ appraisals of risk [18]. Other theories suggest that a combination of trust, intuition, and emotions play a significant role in shaping risk perceptions during conditions of uncertainty [19].

Together, social and individual level processing of risk messages impact behavior and decision-making. On a societal level, a ‘ripple effect’ of amplified risk perceptions can result in fear, stigmatization and aversion behavior spreading far across geographic, temporal and sectoral boundaries, resulting in significant personal, political, and economic impacts [16]. This ripple effect was clearly apparent in the globalized fear response during this outbreak, which spread much further than the disease itself.

### Methods

This qualitative study took place from July 2014 through to January 2015. Ethical approval was obtained from the University of Sheffield School of Health and Related Research. Anonymity has been ensured by de-linking demographic information from pseudonyms used in direct quotes.

### Study setting and participants

The setting of this study is an international one, as participants worked in various locations in Sierra Leone and Liberia, and were subsequently interviewed in their home country or a third country during the post-mission period. Due to the qualitative methodology, non-probability sampling was undertaken [20]. Convenience sampling was used to identify international health care workers who had recently returned from working as front-line health staff in the West African EVD outbreak. Two of the participants were previously known to the researcher from professional networks. Other participants were identified through snowballing, a process in which the researcher identifies participants through contact information given by other participants, and as such utilizes natural social networks [21]. Recruitment was also done through posting notices on internal Médecins Sans Frontières (MSF) Association social media sites. The study limited participation to those international health care workers who worked directly with affected patients, and did not include other non-medical auxiliary staff such as water and sanitation, or management positions who were not in direct contact with patients. We aimed to include males and females, doctors and nurses, and a diversity of nationalities in the sample.

All participants were working for the same international non-governmental organization, MSF, and all but one participant had past work experience with this organization. The majority of participants worked in an Ebola Management Centre (EMC) - a health facility dedicated exclusively to the testing and management of patients acutely ill with EVD. Two of the participants were also charged with community outreach and contact tracing, which took them into local communities. The sample consisted of eleven health care workers with seven different nationalities including Canadian (2), Japanese (2), Nigerian (1), Ugandan (1), Italian (2), Danish (2), and US American (1). The participants were experienced medical professionals, with a mean of 10 years professional experience (range 5–22 years). They consisted of six nurses, four doctors/clinical officer and one public health specialist. The mean age is 35 years old (range 28–46) (see Table 1). Only one participant was married, although several others were in long-term relationships, and only one participant had children.

### Data collection and analysis

The majority of interviews (*n* = 9) were done via Skype, and the remainder (*n* = 2) were done face-to-face in Copenhagen, Denmark. The interviews followed a topic guide, which instigated the interview with a narrative question: “The moment you were offered to work in the Ebola project, what went through your mind?”. This question was followed up with semi-structured questions when needed, exploring the participants’ motivation to work in an Ebola mission, risk perceptions, factors influencing their decision to respond to Ebola, reactions from friends and family as well as the preparedness of the organisation sending them out. This narrative approach provided the advantage of placing greater emphasis on the perspectives of the participant rather than on the researcher’s concerns [20]. The interviews averaged 1 h 50 min in length and took place within two months of participants’ return from West Africa. The interviews were done retrospectively to capture any change in willingness that occurred throughout the experience, including before, during and after the mission.

Analysis of the data was approached using conventional qualitative thematic analysis [22]. Anonymized transcripts were read repeatedly in their entirety, to establish familiarity and to get a holistic view of the data set. The interviews were then analyzed line-by-line and the entire data set was systematically coded for content. A thematic map was developed through the categorization of these initial codes into broader organizing themes, and the organizing themes into global themes. Representative quotations were selected for each main theme discussed. The use of theory to guide analysis was an iterative one. While literature on risk perception had been examined prior to analysis, a specific theoretical framework had not been chosen in advance. Table 2 outlines the development of the global theme from organizing themes, basic themes and primary codes. The basic themes form the structure of our presentation of findings.

**Table 1:** Description of Participants

Identifier/ Interviewee	Age/Sex	Profession	Years of professional experience	Number of Ebola missions	Nationality
1	38/F	Nurse	12	1	Japanese
2	46/M	Doctor	10	2	Nigerian
3	43/F	Nurse	22	2	Japanese
4	39/F	Doctor	10	1	Italian
5	32/F	Nurse	6	1	Danish
6	29/F	Nurse	7	1	Italian
7	36/F	Doctor	10	1	Danish
8	37/M	Clinical officer	13	5	Ugandan
9	29/F	Nurse	7	1	Canadian
10	29/F	Public health specialist	5	2	American
11	28/F	Nurse	8	1	Canadian

**Table 2:** Thematic Analysis: from codes to global theme

Codes	Basic themes	Organizing Theme	Global Theme
- Perception of the risk when deciding to respond - Types of risks perceived - Motivations - Benefits of responding	1.Perceived risks and benefits of responding	Individual determinants of risk perception	Accepting the risk and choosing to respond
- Knowledge or skills - Past experience with risk - Self-efficacy - Demographics	2.Individual characteristics and past experiences as risk modifiers		
- Affect heuristic - Availability heuristic - ‘Othering’ the risk	3.Use of cognitive heuristics in the risk decision		
- Media messages - Influence of media messages on self/family - Other sources of information	4.The influence of media messaging on willingness to respond	Social determinants of risk perception: information sources and their influence on willingness.	
- Communication to/from MSF -Training and preparation - Trust in organisation -Teamwork -Voluntary response	5.The influence of institutional trust and communication on willingness to respond		
- Family - Concerns of family - Impact of family’s opinion - Communication to/from family - Public sentiments - Stigma	6.The influence of family and public on willingness to respond		

## Results

### Perceived risks and benefits of responding

The decision to leave home for West Africa to confront a deadly disease was not a decision that was taken lightly, however many participants described a surprisingly quick and enthusiastic response to the request to go. There was a marked lack of fear and an appraisal of their risk of becoming infected that was minimal or very low:

S: At that time, how did you see your risk of becoming infected?

E: Really low because at that time there weren’t any expats that had become infected. Really, really low at the beginning. And I had a big trust in MSF so I didn’t (laughing), I knew there was a risk, but it was not an issue. (*Emma – nurse*)

M: I didn’t feel fear or anything. I think I just, I wanted to go. That was all.

S: So you weren’t afraid of it?

M: No, not at all. (*Mae – nurse*)

A few participants emphasized that their main fear at this time was not contracting Ebola, but rather the impact their decision would have on their families, and they were more preoccupied about this than their own personal safety:

I got a letter from MSF asking for doctors, and immediately after I got the letter, I thought this is just exactly what I want to do. And then, afterwards, mostly I was thinking that there would be some family members who would not appreciate it very much because they would be very scared. I thought of their fear more than my own. (*Hannah – doctor*)

Oh, the risk of becoming infected. Oh, it wasn’t only about me going for Ebola mission. It was much more than me because I have a family. I mean - my siblings, my father, and of course I know that if I get infected it was going to affect my family as well. I mean they could be stigmatized anyway. I had that in the back of my mind. (*John – doctor*)

Adding to this minimal feeling of risk, there were numerous motivating factors to responding including: recognizing the need; wanting to contribute to a humanitarian crisis; compassion and a duty of care; seeking experience and professional knowledge; and curiosity and a drive to be part of a global event:

I guess I wanted to help. People actually need help, and I can actually do that. So there is actually something about believing in humanitarian work for me. Not just being an adventure, but believing that we should do something to help countries that really need it. (*Anna – nurse*)

I was not scared because I am a doctor, and my duty is to treat people who are suffering, who have medical needs. (*John- doctor*)

### Individual characteristics and past experiences as modifiers of risk perception

Individual level factors seem to be attenuating feelings of risk in this group. Even though most of the participants had not worked with Ebola before, their past professional and life experiences gave them both a sense of duty and feelings of familiarity with what they were going to encounter, such as dealing with deaths or infectious diseases. The majority had previously worked in conflict zones and appear comfortable in risky places. As such, they expressed a strong sense of self-efficacy in being able to cope and as well as a sense of situational control:

I am used to working with parents and children, and children who are dying. So I wasn’t afraid of seeing dead people… But I always felt sure that I could do this without, how do you say, without being too emotionally involved. So, for me, my personality, I felt that it was a task I could do. (*Anna – nurse*)

I mean you look at yourself and you say I’m not stupid. I know I can be a bit crazy but I know that I am very careful. And I have been living alone for ten years in countries that were not my countries, and were not even similar to mine, and I always made it. Having to face different problems and different things. (*Marcia – doctor*)

Interestingly, given their familiarity with risky places, some weighed their risks not as ‘staying home vs. going to an Ebola project’, but rather ‘going to a conflict zone vs. going to an Ebola project’:

Of course I knew there was a risk, there was always going to be a risk. But for me it was not high enough to not go. Because for me, I was thinking, I just went to Bangui and it was a war zone, and there was a grenade exploding in front of my house on the second day, so what is worse? Going to war, or going to a place where there is no war, and you just have to work in another way? So for me it was kind of weighing between. (*Emma – nurse*)

Ebola mission is almost the same as the other missions for me. I mean when I consider the risk of the mission, there is always risk. Kind of, uh, suicide bomb, kidnapping, or robbing, or road accident or whatever, so Ebola can be the same risk as the other missions. (*Mika – nurse*)

### Cognitive heuristics and the decision to respond

Given their self-efficacy, past experience, and the voluntary nature of the risk decision, the predominant emotions on learning they would be going to an Ebola project were positive – including compassion, intellectual curiosity, excitement, and pride:

I was kind of proud. Because it was in September, and it was really big, and I really wanted to know more about that and to be part of that. I was happy. (*Emma –nurse*)

The above quote illustrates the affect heuristic, where positive affect around a risk object or event may result in attenuating feelings of risk [17]. Another heuristic apparent in risk perception is the ‘availability’ heuristic, which involves judging an event as likely or frequent if instances of it are easy to recall [23]. Here, two participants describe how they incorporated this recall into their risk decision:

Before I signed up I was ok with the risk, and ok seeing one of my friends coming back and he was completely healthy, so that gave me a sense of confidence when you know someone who came back healthy. (*Catherine – nurse*)

I don’t know how many expats we’ve sent over since the beginning of the outbreak, but I think it’s more than a couple hundred, maybe even thousands all over the world and you have 3 expats, 23 positive cases [for MSF staff] and 3 of them were expats, so the chances of you getting sick are still pretty low. *(Anna – nurse)*

The above quote also demonstrates a dimension of ‘othering’ occurring, as participants attempt to recall someone similar to themselves who had become infected, to determine their level of risk. There is a feeling that belonging to a certain group (international vs. local, or MSF vs. other organization) will attenuate their risks:

And I read the protocol, and it was also important to me to know those facts about how many people are working, how many got infected. Not as much for the national staff, but for the international staff. (*Anna – nurse*)

When I signed up I was ok with it, and that was in September, and over the course of the month, and I knew the news of the two Americans over the summer from Samaritan’s Purse got infected…and somehow in my mind I thought that Samaritan’s Purse or other non-governmental organisations probably didn’t have any experience with managing Ebola, and who knows what their protocols were, or who knows what their infection control procedures were at work and also where they were living. (*Catherine – nurse*)

For others, the infection of someone close or similar to themselves was a key event in significantly increasing their sense of personal vulnerability. Mika had successfully completed one mission prior to the first expatriate health staff becoming infected, and she describes the shift in risk perception that occurred when someone similar to herself became infected:

I was frustrated during the second mission because we had the first expat nurse, I think she was a nurse, French nurse, who got infected in Liberia. She was the first one. The day she got infected, I arrived in Brussels for the briefing. And I knew that maybe everyone has more risk to get Ebola, because we must have known that we had a risk to be infected, but it was not so strongly real to us, for everyone. But that time, things became more real to everyone. (*Mika – nurse*)

Mae, another nurse, went through a similar experience when a nurse she had been working alongside contracted EVD. Her quote demonstrates both the ‘othering’ of the risk and the risk amplification that occurred when that othering was no longer possible:

But right after that my nurse, local nurse, got infected, and died with Ebola. Actually, after his death, I started feeling, how to say, it is really dangerous mission. Before that, I really didn’t feel it was dangerous. I mean, I really believed I didn’t get Ebola. I thought, that was another world, getting Ebola was in another world. (*Mae- nurse*)

The infections of co-workers strongly increased feelings of risk, and influenced willingness to continue working, in some cases resulting in the evacuation of whole teams of staff. In these moments of crisis, factors that reduced willingness to continue working included: lack of trust in team leadership; exposure occurring in the living quarters rather than the EMC; and fatigue. On the other hand, good communication, trust in the organization and its policies and procedures, trust in coworkers, strong teamwork, and recognizing the voluntary nature of the risk attenuated risk feelings and fostered an ongoing willingness to work.

### The influence of institutional trust and communication on willingness to respond

Trust in the organisation, and regular communication from the organisation, were key risk attenuators, and countered some of the fearful messages coming from the media. Many participants stated that their low level of fear and subsequent decision to respond was due in part to their trust in MSF in keeping them safe. It is notable that 10 out of 11 participants had prior experience working with MSF, and this familiarity engendered trust:

So most of my information was reading the protocols, and the story of Ebola – when did we discover it, how long has MSF been working with it. For me it was really important to know that MSF had experience in it. That made me feel secure working there. (*Anna – nurse*)

I thought for my own risk, that when working for a professional organization like MSF, I thought the risk for myself would be very low because I was sure they would have all the right materials and procedures to make it very safe. (*Hannah – doctor*)

The provision by the organization of information in advance, including trainings and briefing materials, as well as timely messages around cases of staff infection were key in attenuating feelings of risk that may have been perpetrated in the media:

You know what, MSF is really good at keeping us informed. Their HR department sends out lots of emails to people who are either on an Ebola mission or preparing to go for an Ebola mission, especially around cases where staff were infected, and so I knew that for every contamination of expat or national staff there is an investigation that is conducted to see what went wrong, how did that person get infected. (*Catherine – nurse*)

### Influence of the media on willingness to respond

Images, such as those from the media, may evoke a strong affective response, either positive or negative, and subsequently influence perceptions of risk. Interestingly, while media images of health workers in biohazard suits may have evoked fearful emotions in the general public, the same media images evoked a sense of curiosity and an affective draw in some participants. Media images also reinforced a sense of need and urgency:

…at the same time I was studying biology and many things related to tropical medicine, and I was following the news every single day and I felt like I needed to go there…And when I saw the news and people wearing the PPE [personal protective equipment] and working in isolation area, I felt really like really going and working there. (*Mae –nurse*)

I vaguely remember watching a news report about an Ebola outbreak and seeing the doctors and nurses on the screen and thinking, “I want to do that”. (*Allison – public health*)

### Family and public perceptions and willingness to respond

Media coverage resulted in an opposite dynamic in family members. Without the same professional knowledge and duty of care, experience in risky situations, or access to other sources of information, the media coverage appears to have taken a dominant role in forming the risk perception in family members, and evoked a fear reaction in some:

They were afraid for me. They were more afraid than I was. Maybe because I know MSF, I know how it works, I know their rules, and I know at the end, that the virus doesn’t run after people and jump on you. So I was not like that comfortable that my family and friends were really afraid from these crazy things on the t.v. Like people buying these things, this paranoia actually. They were much, much more afraid than I was. (*Emma – nurse*)

Of course, because I had been following the outbreak since the beginning because it is something I had been very interested in, and of course and if you only get your information from the media, you may be more scared of going than if you also have some more scientific information, so I also think that’s why my family members were more afraid than I was, because when you only see bad stories from the media, you may perceive the risk as larger than it actually is. (*Hannah – doctor*)

According to participants, the impact of this fear reaction of family members became more significant over time as the outbreak received more media coverage. Several families who had not previously objected to their loved one working in an Ebola project early in the outbreak, later changed their minds as media coverage intensified. This risk amplification within family members ultimately resulted in a reduction in willingness to respond:

Well actually I got offer one more time, for Ebola mission, but I didn’t go because of my family…My family didn’t allow me to go this time. So I couldn’t go….and I didn’t want to let my Dad and Mom cry again for me. (Mae – nurse)

Public perceptions may also play a significant role in the health care worker’s willingness to respond. Negative remarks against the returning health workers from people in immediate social circles as well as the public on social media resulted in many participants experiencing feelings of distress and stigmatization upon their return home:

Like me, I got many opinions and comments from people. It made me really down. I’m ok, because I came back, but if someone wants to go to Sierra Leone or Liberia to help people, it might be making people not want to go. It’s really cruel I feel. We are not prisoners you know. We didn’t commit crimes. (Mae- nurse)

## Discussion

Given the importance of front-line health care workers in providing care during infectious disease outbreaks, it is critical to better understand the factors that contribute to their willingness to respond, and how risk perception may influence such a decision. While fear of contagion of self and family is commonly reported by health care staff in epidemics [9, 24–27], this study found the perceived risk of becoming infected was significantly modified by numerous individual and social factors.

Previous studies have highlighted that both the acknowledgment of the threat and a sense of efficacy in carrying out one’s role in the response - a ‘concerned and confident’ profile - are important determinants of willingness [10, 11, 28]. Our study supports the role of self-efficacy and confidence in contributing to willingness but found numerous other contributing factors. Past experience, humanitarian ethos, duty of care, curiosity, and trust emerged as risk attenuators at the individual level. The filtering and evaluation of competing messages from the media, MSF, and friends and family also impacted feelings of risk. The “affective” impact of media reports and images of biohazard suits may have created fear and dread in family members and members of the public resulting in feelings of “high risk”, however the participants were not impacted in the same way. Instead, they were attracted by curiosity and a sense of need (which they could effectively meet) arising from media images. This affect heuristic, coupled with other heuristics, including ‘othering’ the risk, may have contributed to an advance assessment of the risk as “low”. The regular communication of factual information from MSF further helped to inform participants and counter risk messages coming from the media.

This study has also highlighted organizational trust as a key factor in attenuating feelings of risk and contributing to a willingness to respond. This is in line with Ives’s finding that lack of trust in the NHS may deter health workers from responding during an influenza pandemic [14]. Marjanovic, Greenglass and Coffey found that vigour, organisational support and trust in equipment and infection control predicted lower levels of avoidance behaviour, emotional exhaustion and anger in a survey of nurses who had worked with SARS in Toronto [29]. Trust in the organisation was not only important to the initial agreement to respond. Elsewhere we have reported on how trust in the equipment, policies and procedures and team leadership were key factors in supporting ongoing willingness following moments of crisis, such as the infection of co-workers, as well as for successful day-to-day coping [30].

Trust has been shown to play a significant role in risk perception with both general trust (the belief that others can be relied upon) and general confidence (the conviction that things are under control) able to reduce perceived risks [31]. It has also been argued that who we trust is less related to technical competence and more related to similarities in values [31–33], particularly when there is a lack of technical knowledge on the specific risk. In our study, trust in the organization seems to be related to MSF having the technical knowledge, experience and sufficient equipment to keep them safe, as well as a ‘salient value similarity’ [32] in terms of humanitarian ethos and “believing in MSF” as one participant stated. This value-based trust, however, may also lead to what is referred to as identity-protective cognition - the need to conform to the risk culture of the key members of one’s group. Individuals tend to adopt beliefs common to their group membership and are more likely to reject information from “outsiders” that counter the common beliefs of the group [34]. Such a trust may have allowed participants to accept MSF’s assessment of the risk in advance of experiencing it themselves, and allowed them enough peace of mind to continue working when face-to-face with contagion. This institutional trust may further explain why fearful media images had less direct impact on the health workers than they did on family, friends, and the public.

### Implications for practice and future research

#### Fostering trust

Trust was found to be an important component of willingness to respond in this study. While preparation of staff for such high-risk missions must include technical trainings in order to build role familiarity and confidence, efforts must be also made to build and foster institutional trust through providing a safe workplace, team building, strong leadership and clear communication. Reinforcing the voluntary nature of the work by not instituting mandatory response or activities may also increase health workers comfort and decrease feelings of risk. As well, all efforts must be made to ensure a clear and balanced message of risk be provided not only in advance but throughout the work. It is notable that these responders perceived their personal risk to be low prior to entering the epicenter, and only after the disease infected those close to them did their perception of personal risk shift. Organisations should anticipate such shifting in risk appraisal and be prepared with a clear and supportive institutional response.

#### Taking care of health workers

Skilled and experienced health care workers who are willing and able to work in higher risk settings are rare, and their wellbeing must be considered even after they have returned home. Given the multitude of stressors involved in this type of work, proactive psychosocial support must be provided before, during, and after the response. This is in addition to the provision of an initial debriefing after completion of a mission. The small body of evidence that examined the longer-term psychological impacts of responding to the SARS outbreak indicates an increased risk for post-traumatic stress disorder [35–37] following this type of work and further highlights the importance of ongoing psychosocial support that should continue through the post-response period.

Similar to the findings of Gershon et al. [13] of distress and transition difficulties upon returning home, this study found negative public sentiment towards returning health care workers, influenced by media reports and confusing public health policies around quarantine, created distress in participants. This has the potential to reduce willingness to respond in future outbreaks. Clear and consistent messaging from both political and public health bodies may go a long way in tempering public panic and creating an atmosphere of support for health workers.

#### Taking care of families

Despite being mature and independent professionals, the fear and worry of family members had a strong impact on participants in this study, and for several reduced their willingness to continue working during the outbreak. While participants benefited from regular information and communication from MSF, there seemed to be minimal to no proactive communication between the organization and family members. Supporting family members, through proactive and ongoing dialogue with family by the organization, including informational and psychosocial support, may help to overcome the negative influence of media reports, and ultimately benefit the response capacity.

#### Limitations and future research

This study is limited in that it focused specifically on the subset of ‘willing’ responders, and was limited to one international organization. Further, it was restricted to a relatively small number of international health workers and did not explore experiences of local health staff, which itself is an important area of future study. It would be valuable for future research to explore the differences in risk perceptions between those who chose to respond and those who chose not to, in order to further elucidate the influences of specific individual and social determinants of risk perception. As this study found communication from MSF attenuated feelings of risk, a comparative discourse analysis of risk communication coming multiple social actors such as the media, the government, and bodies such as MSF, WHO, and Centre for Disease Control (CDC) would help to evaluate the role of communication strategies on willingness to respond.

## Conclusions

While risk is an abstract and socially constructed phenomenon, risk perceptions have very real consequences for public health response capacity. We have outlined key individual and social level modifiers of risk perception that influenced the participants’ willingness to respond to the West Africa Ebola outbreak. Risk perception theories have provided useful explanatory mechanisms to explore risk perception on both individual and social levels, and this study has highlighted the importance of the role of trust in this process. Overall, this study has shown that an understanding of risk perception of health workers and their families, institutions, and the public, while complex and interdependent, are each crucial to understand for an effective public health response to epidemics, and as such should be taken into consideration in future program planning and research.

## Abbreviations

CDC: Centre for Disease Control
EMC: Ebola Management Centre
EVD: Ebola Virus Disease
HCWs: Health Care Workers
HIV: Human Immunodeficiency Virus
MSF: Médecins Sans Frontières
NGOs: Non-Governmental Organisations
PPE: Personal Protective Equipment
SARF: Social Amplification of Risk Framework
SARS: Severe Acute Respiratory Syndrome
WHO: World Health Organisation.
